# The effect of psychosocial stress on single mothers’ smoking

**DOI:** 10.1186/1471-2458-13-1125

**Published:** 2013-12-05

**Authors:** Stefanie Sperlich, Mercy Nyambura Maina, Dorothee Noeres

**Affiliations:** 1 Hannover Medical School, Medical Sociology Unit, Carl-Neuberg-Str. 1, 30625 Hannover, Germany

**Keywords:** Single mothers, Smoking rate, Psychosocial stress, Mediation

## Abstract

**Background:**

Evidence suggests an increased risk of smoking among single mothers as compared to their cohabitating counterparts. This article examines the role of psychosocial stress in mediating the relationship between single motherhood and smoking.

**Methods:**

Data were derived from a cross-sectional population based sample of German women (n = 3129) with underage children (0–18 years of age). Perceived stress was measured with 13 items covering socioeconomic as well as family- and parenting-related stressors. According to Baron and Kenny (1986) a series of logistic regression models was applied to investigate the role of psychosocial stress as a mediator on the relationship between single motherhood and smoking.

**Results:**

About 44.0% of single mothers smoked daily, whereas only 26.2% of cohabitating mothers did. Single mothers reported more stress related to their economic situation, occupation and family than partnered mothers. Out of the original 13 stressors only 'conflicts with the partner or ex-partner’ and 'financial worries’ remained significant in explaining single mothers’ higher risk of smoking. Against expectation, stress due to household requirements and family demands was associated with lower odds of single mothers’ smoking. After controlling for psychosocial stress, the odds ratio of single mothers’ moderate smoking (< 20 cig./day) decreased slightly from 1.75 to 1.66 (explained fraction XF = 12.0%) and with respect to heavy smoking (≥ 20 cig./day) more pronounced from 2.56 to 2.01 (XF = 35.3%).

**Conclusions:**

It can be stated that single mothers’ heavy more than moderate smoking appeared to be mediated by perceived psychosocial stress. Out of all stressors considered, financial worries were of paramount significance in explaining single mothers’ heavy smoking while some family-related stressors rather appeared to keep single mothers from smoking. Overall, a higher stress exposure explains partly but not sufficiently single mothers’ increased smoking rates.

## Background

In Germany every fifth family with a minor child is headed by a single parent, 90% of them are single mothers [1]. There is consistent evidence in Western countries that smoking prevalence in single mothers is higher than in married and cohabitating mothers [2-6]. A common explanation from a medical-sociological point of view would be that single mothers’ increased tobacco use is due to their higher risk of socioeconomic disadvantages. A large body of literature has demonstrated that single mothers are more likely to be employed in lower status and lower paying jobs and to be at higher risks of poverty, poor psychosocial working conditions, work-family conflicts and financial hardships [7-10].

The conceptual framework of 'the social patterning of smoking’ [11,12] suggests that individuals in a low socio-economic position experience more psychosocial stress and use smoking as a way of coping with difficult and stressful circumstances in order to come to terms with negative emotions. Numerous studies have confirmed this assumption revealing that stress is more often reported by smokers than non-smokers. Smokers experience higher levels of occupational, marital and financial stress and are also more affected by adverse life events such as divorce and unemployment [13]. Cohen et al. [14] found that four psychosocial factors were associated with women’s smoking: a history of depression, marital conflicts, undesirable life events, and full time employment.

**Figure 1:**
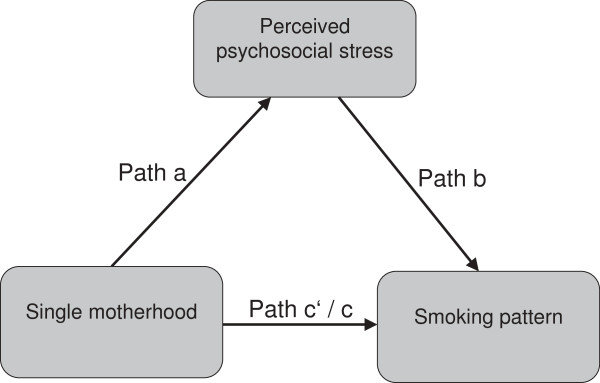
**The Mediator Model of single motherhood on moderate and heavy smoking patterns.** Path c indicates the total, c’ the direct and path a * b the indirect effect of X on Y.

Psychosocial stress has been linked to the risks of smoking across all stages of smoking, that is, initiation, maintenance and relapse [13]. The strongest evidence has been found for the impact of financial hardship on smoking behaviour, holding in particular for single mothers [4,15-17]. However, Siapush et al. [6] pointed out that the association between single motherhood and smoking is not fully explained by socioeconomic disadvantages. In contrast to Dorsett and Marsh [18] they found a strong 'lone mother effect’ on smoking after controlling for socioeconomic status. They argued that there are other factors closely related to the experience of being a lone mother, such as social isolation and loneliness, which play an important role in explaining single mothers’ smoking behaviour. Jun and Arcevedo-Garcia [3] criticised that studies on single mothers rarely examined the influence of parenting on smoking. They could demonstrate that child care responsibilities significantly contributed to smoking patterns among white single mothers. All in all, previous studies suggest that psychosocial stress plays an important role in explaining smoking among single mothers. However, this evidence is mainly based on the effects of socio-economic hardships while less is known about the impact of parenting-related stressors on single mothers’ smoking. Similarly, there is little knowledge on how the impact of stress differs with respect to moderate and heavy smoking patterns. Against this background, this study aims to analyze the relevance of various stressors in explaining moderate as well as heavy smoking habits among single mothers. In more detail, the paper addresses the following questions (see Figure 1):

1. How does perceived psychosocial stress differ between single and partnered mothers?

2. Does psychosocial stress account for moderate and heavy smoking pattern and if so, does its effect hold for both single and partnered mothers?

3. Is single mothers’ higher smoking rate mediated by psychosocial stress?

## Methods

### Sample

The sample consists of 3129 mothers with underage children (0–18 years of age) living in Germany. The cross-sectional survey was conducted by TNS Healthcare on behalf of the Department of Medical Sociology at Hannover Medical School. The ethics committee of Hannover Medical School approved the study. Data were collected in 2009 by means of a mail survey. The sample was derived from the Healthcare Access Panel comprising 75.000 households in total and 27.038 households with women having underage children. The Healthcare Access Panel is composed of respondents who have given their general consent to participate in surveys. Based upon an estimated response rate of 50 percent and a targeted case number of 2.500 mothers a total of 5.000 German mothers was selected randomly out of the panel. The gross sample was drawn according to predefined quotas, i.e. age of mother and youngest child, school education, marital status and number of children. The initial case number of young mothers (≤ 25 years) had to be completed by another 107 cases in order to meet the quota. Of these 5107 mothers 3183 participated in the survey, corresponding to a return rate of 62.3%. A total of 54 mothers was excluded subsequently because they did not meet the inclusion criteria (in particular youngest child was > 18 years of age). The final sample consisted of 3129 mothers with 506 (16.8%) out of them were single mothers. The number of missing values with respect to the key variables 'single vs. partnered mothers’ (3.6%) as well as smoking pattern (0.8%) is relatively small. Hence, concerning these variables selection bias due to non-response are negligible. The sample was selected in proportion to the distributions of German federal states, school education, mother’s age, and number of children. Subsequently, the cases were weighted in order to obtain data representative for German mothers (Table 1).

### Smoking pattern

Smoking pattern was assessed by asking the women whether they currently smoke cigarettes. Pipe smoking was not assessed. The outcome variable 'current smoking status’ includes three categories: (1) smoking daily (2) smoking occasionally (not daily) and (3) not smoking at all. If the women answered 'smoking daily’, it was asked how many cigarettes they smoked per day. The literature on smoking has no widely accepted criterion for separating light from heavy smokers. In line with Billings and Moos [19] we selected 20 cigarettes per day as a level at which tobacco dependence was probable and used this as a threshold for heavy smoking. The variable 'smoking pattern’ was then defined as a three category outcome variable with non-smoking (currently not smoking at all), moderate (1–19 cig./day) and heavy smoking (≥ 20 cig./day).

### Social and family related factors

Socioeconomic status was measured with the following variables: school education, employment status and income. We categorized the household income based on needs-adjusted equivalence scales into three categories: (1) < 60% of the German median income, (2) between 60% and 100% of the German median income, and (3) above 100% of the German median income [20]. Single mothers are defined as mothers with at least one underage child living in the household without a partner, i.e. mothers cohabiting with a partner were not defined as single mothers but as 'partnered mothers’.

### Psychosocial stress

*Perceived psychosocial stress* was measured by the questionnaire 'Parental Stress’ (13 items) which proved to be relevant for mothers caring for young children [21,22]. Each item has five categories, ranging from 1 (not at all distressed/not applicable) to 5 (very strongly distressed). Mothers were asked how much they considered themselves as to be distressed with respect to the following stressors: (1) financial worries, (2) work-related adversity (including also stress due to unemployment), (3) partner-related problems (conflicts with current or former partner) (4) household requirements, (5) family demands (i.e. permanent availability for the family), (6) sole responsibility for children, (7) having a disabled or a chronically ill child, (8) child-related stress (e.g. child-rearing difficulties, conflicts in mother-child-interactions, problems at school), (9) having a family member in need of care (partner or a parent), (10) balancing job and family demands, (11) conflicts with other family members (e.g. parents, parents in law), (12) unwanted living alone/loneliness and (13) stress due to lack of appreciation (less recognition of mother-role) (see Additional file 1).

### Further instruments

Due to strong associations between smoking and mental health [23], we used the subscale 'depression’ from the Hospital Anxiety and Depression Scale German Version (HADS-D) [24] in order to control for this possible confounder in our multivariate analysis. The subscale contains seven items, each ranging from 0 to 3. We used the sum score ranging from 0 to 21.

### Statistical analyses

According to Baron and Kenny [25] psychosocial stress acts as a mediator for the relationship between single motherhood and smoking when the following conditions are met:

1. Being a single mother is associated with significantly increased rates of moderate and heavy smoking (Figure 1, path c). This condition was tested by regressing the dependent variables (moderate and heavy smoking) on the independent variable (single motherhood).

2. Being a single mother is associated with higher levels of perceived stress in daily life (Figure 1, path a). This presumption was examined by regressing the mediators (psychosocial stress) on the independent variable (single motherhood).

3. High levels of perceived stress in daily life are associated with higher rates of moderate as well as heavy smoking pattern (Figure 1, path b). This was analyzed by regressing the dependent variables (moderate and heavy smoking pattern) on the mediators (psychosocial stress). In order to analyze whether the effects of stress on smoking hold for both single and partnered mothers, interaction effects between single motherhood and psychosocial stress were computed.

4. The effect of single motherhood on moderate and heavy smoking pattern must be smaller in presence of psychosocial stress (Figure 1, path c’ < path c). This was tested by regressing the dependent variables (moderate and heavy smoking pattern) on both the independent variable (single motherhood) and psychosocial stress (mediators). A stepwise logistic regression analysis (method: forward selection, likelihood quotient) with two blocks was computed for moderate as well as heavy smoking pattern. Block 1 as the baseline model contains the total effect of single motherhood adjusted for maternal age, age of youngest child and depression (HADS-D). Block 2 adds the stressors meeting the conditions to establish mediation (see above). The decline in odds ratios from block 1 to 2 reflects the influence of psychosocial stress on single mothers’ smoking pattern. The percentage of single mothers’ odds for moderate and heavy smoking explained by psychosocial stress (explained fractions XF) was calculated using the common formula [26]: XF = (OR baseline model – OR extended model)/((OR baseline model) -1).

All conditions were tested using binary logistic regression analyses with the following dichotomous variables: Psychosocial stress = high stress (categories 4 and 5 of response format) vs. low stress (categories 1 to 3 of response format), single motherhood = yes vs. no, moderate/heavy smoking = yes vs. not smoking. For testing conditions 1 to 3, odds ratios were adjusted for maternal age and age of the youngest child. Pearson’s χ^2^ test was used for testing the statistical significance of differences between single and partnered mothers with respect to sample characteristics. Statistical analyses were performed with SPSS version 19.0.

## Results

### Comparison of single and partnered mothers

Compared to partnered mothers (mean age 39.2 ± 6.5), single mothers (mean age 38.6 ± 6.9) were more likely to have only one child and were less likely to have an infant child (0 to 2 years). With respect to their socioeconomic status single mothers showed lower educational levels and they were at higher risk of poverty (< 60% median income). In addition, single mothers were more likely to be full-time employed as well as unemployed and looking for a job compared to partnered mothers.

**Table 1:** Sample characteristics of single and partnered mothers

	**Single mothers n = 506**		**Partnered mothers n = 2511**		**Chi**^**2**^
	**n**	**%**	**n**	**%**	**p value**
**Age (years)**					0.731
20-29	49	9.8	226	9.1	
30-39	220	44.0	1068	42.8	
40-49	207	41.4	1096	43.9	
50-59	24	4.8	106	4.2	
Missings	6		15		
**Age of the youngest child (years)**					<0.001
0-2	30	6.0	437	17.6	
3-5	72	14.4	403	16.2	
6-11	187	37.5	789	31.7	
12-15	139	27.9	527	21.2	
16-18	71	14.2	331	13.3	
Missings	7		24		
**Number of children**					<0.001
1	293	60.7	842	35.8	
2	145	30.0	1099	46.7	
3 +	45	9.3	410	17.4	
Missings	23		160		
**Years of school education**					0.014
≤ 9	187	34.4	772	31.0	
10	181	36.2	1037	41.6	
≥ 12	132	26.4	682	27.4	
Missings	6		20		
**Income**					<0.001
< 60% median	291	60.0	508	23.7	
< 100% median	151	31.1	893	41.7	
>100% median	43	8.9	738	34.5	
Missings	21		372		
**Employment status**					<0.001
Work ≤ 19 hours/week	71	14.5	442	23.6	
Work 20–37 hours/week	200	40.8	922	37.2	
Work ≥ 38 hours/week	124	25.3	394	15.9	
Housewife/Maternity leave	37	7.6	517	20.9	
Unemployed	44	9.0	48	1.9	
Early retirement	14	2.9	12	0.5	
Missings	16		34		

### Effect of single motherhood on smoking

The percentage of single mothers who smoked daily was 44.0% compared to 26.2% among partnered mothers (Table 2). This corresponds to an odds ratio of OR = 2.11 for daily smoking among single mothers. Most pronounced is the difference in smoking pattern with respect to heavy smoking: nearly every fifth single mother (19.2%) smoked at least 20 cigarettes a day, whereas this applied only to 8.4% of partnered mothers (OR = 2.87). Also moderate smoking patterns (OR = 1.73) as well as occasional smoking (OR = 1.69) were higher in single than in partnered mothers.

**Table 2:** Effect of single motherhood on smoking

	**Single mothers**		**Partnered mothers**		**Single motherhood**	
**Smoking status (current)**	**n**	**%**	**n**	**%**	**OR**	**CI 95%**
Daily smoking (at least 1 cig./day)	220	44.0	654	26.2	**2.11**	**1.70-2.60**
Heavy smoking (≥ 20 cig./day)	96	19.2	211	8.4	**2.87**	**2.16-3.81**
Moderate smoking (1–19 cig./day)	124	24.8	443	17.7	**1.73**	**1.36-2.20**
Occasional smoking (not daily)	34	6.8	126	5.0	**1.69**	**1.20-2.56**
Non-smoking	246	49.2	1719	68.8	Ref.	

Notes: Results of logistic regression analysis adjusted for mother’s age and age of youngest child. Single mothers (n = 506), partnered mothers (n = 2511). Reference category 'single motherhood’ = partnered mothers. 'Ref.’ means reference category. Bold values indicate significant effects.

### Effect of single motherhood on perceived psychosocial stress

Single mothers perceived significantly higher stress levels as compared to their cohabitating counterparts (Table 3). This holds for structural problems ('financial worries’ and 'career-related stress’) as well as stress related to parenting ('child-rearing difficulties), marital difficulties ('conflicts with the partner or former partner’) and family-related stress ('sole responsibility for the children’, 'household requirements’, 'family demands’, 'balancing family and job demands’ and 'little recognition of family work’). The difference between single and partnered mothers was most pronounced with respect to the stressors 'unwanted living alone/loneliness’ (OR = 19.49), 'sole responsibility for the child/ren’ (OR = 8.03), 'financial worries’ (OR = 3.55) and stress related to 'career situation/unemployment’ (OR = 2.89). No significant differences in stress levels between single and partnered mothers were found with respect to having 'a disabled or chronically ill child’, stress caused by 'a family member in need of care’ and stress related to 'conflicts with other family members’.

**Table 3:** Effect of single motherhood on perceived psychosocial stress

	**Single mothers**		**Partnered mothers**		**Single motherhood**	
**High psychosocial stress due to….**	**%**	**n**	**%**	**n**	**OR**	**CI 95%**
financial worries	51.9	261	23.1	575	**3.55**	**2.89-3.58**
career situation/unemployment	38.5	193	17.7	441	**2.89**	**2.34-3.58**
conflicts with the partner or ex-partner	29.4	148	13.9	346	**2.71**	**2.15-3.41**
household requirements	29.5	148	25.0	623	**1.45**	**1.17-1.81**
family demands	34.1	171	29.9	726	**1.51**	**1.22-1.86**
sole responsibility for the child/ren	56.4	238	15.1	376	**8.03**	**6.48-9.99**
a disabled or chronically ill child	5.8	29	4.6	114	1.30	0.85-2.00
child-rearing difficulties	28.3	142	17.2	428	**1.91**	**1.53-2.40**
a family member in need of care	6.0	30	6.5	160	0.93	0.62-1.41
balancing family and job demands	27.5	139	15.3	380	**2.36**	**1.82-2.90**
conflicts with other family members	12.0	60	11.8	294	1.03	0.76-1.39
unwanted living alone/loneliness	31.5	159	2.3	58	**19.49**	**13.94-27.24**
little recognition of family work	25.6	129	15.8	354	**1.88**	**1.48-2.38**

Notes: Results of logistic regression analysis adjusted for mother’s age and age of youngest child. High psychosocial stress = categories 4 and 5 of response format (reference categories = 1 to 3), reference category of 'single motherhood’ = partnered mothers. Bold values indicate significant effects.

### Effects of perceived psychosocial stress on moderate and heavy smoking pattern

The first four stressors displayed in Table 4, namely 'financial worries’, stress due to 'career situation/unemployment’, 'conflicts with the partner or ex-partner’ and 'sole responsibility for the children’ were associated with increased rates of moderate as well as heavy smoking. Odds ratios were more pronounced for the outcome 'heavy smoking pattern’ (OR ranging between 1.63 and 2.54) as compared to 'moderate smoking pattern’ (OR ranging between 1.22 and 1.47). The next five stressors showed a significant effect on heavy, but not on moderate smoking habits. This was the case for 'childrearing difficulties’ (OR = 1.54), 'unwanted living alone/loneliness’ (OR = 2.99), 'little recognition of family work’ (OR = 2.19), 'a family member in need of care’ (OR = 2.10) and 'conflicts with other family members’ (OR = 1.60). The last four stressors showed no significant effect on smoking, neither on moderate nor on heavy smoking pattern. This applied to 'household requirements’, 'family demands’, 'a disabled or chronically ill child’ and 'balancing family and job demands’.

**Table 4:** Effects of perceived psychosocial stress on moderate and heavy smoking pattern

	**Non- smoking**		**Moderate smoking**		**Heavy smoking**		**Moderate smoking**		**Heavy smoking**	
**High psychosocial stress due to…**	**%**	**n**	**%**	**n**	**%**	**n**	**OR**	**CI 95%**	**OR**	**CI 95%**
financial worries	23.5	470	33.3	197	44.8	147	1.22	0.78-1.89	**2.54**	**1.53-4.20**
career situation/unemployment	18.3	363	24.4	145	31.9	105	**1.39**	**1.06-1.81**	**1.64**	**1.15-2.33**
conflicts with the partner or ex-partner	14.5	290	19.0	113	22.4	73	**1.47**	**1.10-1.97**	**1.69**	**1.15-2.49**
sole responsibility for the child/ren	19.9	395	25.4	151	34.3	113	**1.47**	**1.11-1.95**	**1.63**	**1.12-2.38**
child-rearing difficulties	18.3	361	18.4	109	27.8	91	1.02	0.76-1.35	**1.54**	**1.08-2.19**
unwanted living alone/loneliness	6.1	121	6.4	38	16.3	53	1.14	0.55-2.34	**2.99**	**1.48-6.04**
little recognition of family work	16.2	324	15.8	94	27.2	89	1.13	0.84-1.52	**2.19**	**1.55-3.09**
a family member in need of care	6.0	118	4.9	29	11.0	36	0.96	0.61-1.52	**2.10**	**1.29-3.43**
conflicts with other family members	11.4	226	10.6	63	16.1	52	1.16	0.84-1.60	**1.60**	**1.06-2.41**
household requirements	26.3	523	22.3	132	27.7	91	1.07	0.84-1.37	1.19	0.85-1.66
family demands	29.8	593	29.4	175	29.5	97	1.27	1.00-1.60	1.29	0.93-1.78
a disabled or chronically ill child	4.8	95	4.7	28	5.8	19	1.12	0.69-1.83	0.86	0.41-1.79
balancing family and job demands	16.9	336	16.4	97	21.0	69	1.23	0.92-1.64	1.23	0.83-1.83

Notes: Results of logistic regression analysis adjusted for single motherhood, interaction term between single motherhood and stressors, mother’s age and age of youngest child. Moderate smoking = < 20 cig./day, heavy smoking = ≥ 20 cig./day (reference category = non-smoking), high psychosocial stress = categories 4 and 5 of response format (reference categories = 1 to 3), OR = odds ratio, CI 95% = 95% confidence interval. Bold values indicate significant effects.

### Interaction effects between single motherhood and stress on smoking

In order to analyze if the observed relationships between stress and smoking held for both single as well as partnered mothers, interaction effects between single motherhood and stress on smoking were computed by means of logistic regression analysis (Table 5). Most of the stressors effecting moderate as well as heavy smoking pattern (Table 4) showed no significant interaction with single motherhood. The exception was 'sole responsibility for the child/ren’ with an OR = 0.46, indicating that this stressor is significantly less relevant in explaining single mothers’ moderate smoking as compared to partnered mothers. As indicated by odds ratios below 1 also the effects of 'unwanted living alone/loneliness’ and 'little recognition of family work’ on heavy smoking (Table 4) revealed to be less important in single mothers. Similarly, family-related stressors, namely 'conflicts with other family members’, 'household requirements’, 'family demands’, 'a disabled or chronically ill child’ and 'balancing family and job demands’ showed significant interaction terms, indicating that they were less applicable in explaining single mothers’ smoking, in particular their moderate smoking pattern.

**Table 5:** Interaction effects between single motherhood and stress on moderate and heavy smoking pattern

	**Moderate smoking**		**Heavy smoking**	
**High psychosocial stress due to…**	**OR**	**CI 95%**	**OR**	**CI 95%**
financial worries * single motherhood	1.37	0.82-2.27	0.88	0.48-1.59
career situation/unemployment * single motherhood	0.76	0.45-1.29	1.02	0.56-1.87
conflicts with the partner or ex-partner * single motherhood	0.79	0.44-1.39	0.82	0.43-1.56
sole responsibility for the child/ren * single motherhood	**0.46**	**0.27-0.78**	0.83	0.44-1.54
child-rearing difficulties * single motherhood	0.74	0.41-1.33	0.92	0.49-1.70
unwanted living alone/loneliness * single motherhood	**0.39**	**0.16-0.97**	**0.40**	**0.17-0.95**
little recognition of family work * single motherhood	**0.47**	**0.25-0.89**	**0.52**	**0.27-0.97**
a family member in need of care * single motherhood	0.60	0.18-2.02	0.91	0.33-2.45
conflicts with other family members * single motherhood	**0.37**	**0.14-0.96**	0.83	0.37-1.84
household requirements * single motherhood	**0.32**	**0.18-0.59**	0.66	0.36-1.22
family demands * single motherhood	**0.45**	**0.26-0.76**	**0.49**	**0.27-0.90**
a disabled or chronically ill child * single motherhood	**0.18**	**0.04-0.83**	1.08	0.34-3.44
balancing family and job demands * single motherhood	**0.33**	**0.18-0.62**	0.85	0.44-1.62

Notes: Results of logistic regression analysis adjusted for single motherhood (main effect), mother’s age and age of youngest child. Bold values indicate significant effects. See Table 3 for explanations of abbreviations.

### Impact of psychosocial stress on mothers’ smoking status

Finally, we examined the extent to which single mothers’ higher smoking rates could be attributed to their higher stress levels using forward stepwise logistic regression analysis (Table 6). Our analysis included all psychosocial stressors showing a significant association with single motherhood (Table 3) as well as smoking (Table 4) in the predicted direction. Stressors showing a significant interaction with single motherhood that point in the opposite direction of decreased odds of smoking in single mothers ('Table 5) were not included in the analysis. With respect to moderate smoking the conditions for mediation were only met in case of 'conflicts with the partner or ex-partner’ and stress due to 'career situation/unemployment’. With respect to heavy smoking this holds to 'financial worries’, 'stress due to 'career situation/unemployment’, 'conflicts with the partner or ex-partner’, 'sole responsibility for the children’ and 'child-rearing difficulties’.

**Table 6:** Changes in odds ratios for smoking in single mothers after controlling for psychosocial stress

	**Block 1**^**1**^			**Block 2**			
**Moderate smoking**	**OR**	**KI 95%**	**R**^**2**^	**OR**	**KI 95%**	**XF**	**R**^**2**^
**single motherhood (yes)**	**1.75**	**1.35-2.25**	**.043**	**1.66**	**1.28-2.15**	**12.0%**	**.051**
**conflicts with the partner or ex-partner**							
not at all/not applicable				Ref.			
slightly				0.91	0.71-1.17		
moderately				**1.37**	**1.02-1.83**		
strongly				**1.45**	**1.01-2.08**		
very strongly				**1.58**	**1.07-2.35**		
**Heavy smoking**							
**single motherhood (yes)**	**2.56**	**1.90-3.45**	**.097**	**2.01**	**1.47-2.75**	**35.3%**	**.131**
**financial worries**							
not at all/not applicable				Ref.			
slightly				1.34	0.86-2.10		
moderately				**1.78**	**1.15-2.77**		
strongly				**1.92**	**1.18-3.12**		
very strongly				**4.33**	**2.67-7.01**		

Notes: ^1^Results of stepwise logistic regression analysis adjusted for mother’s age, depression (mean score single mothers: 8.59 ± 4.05 and partnered mothers: 7.23 ± 3.79) and age of youngest child, XF = percentage of smoking explained by the stressor, Pseudo R^2^ = Nagelkerke. Reference category is indicated by 'Ref.’. Bold values indicate significant effects.

Block 1 displayed single mothers’ odds of moderate (OR = 1.75) and heavy smoking pattern (OR = 2.56) adjusted for mothers’ age, age of youngest child and depression. Stepwise regression (Block 2) revealed that 'conflicts with the partner or ex-partner’ is the only significant predictor in explaining moderate smoking pattern, while stress due to 'career situation/unemployment’ failed to reach statistical significance in Block 2. Odds of moderate smoking continuously increased with stress level from OR = 0.91 ('slightly distressed due to 'conflicts with the partner or ex-partner’) to OR = 1.58 ('very strongly distressed’). By entering this stressor in the model, odds ratios for single mothers’ moderate smoking pattern decreased slightly from 1.75 to 1.66. This corresponded to a change in the odds ratios of 12.0%.

'Financial worries’ proved to be most important in predicting single mothers’ heavy smoking pattern. Odds of heavy smoking climbed up to OR = 4.33 when women reported to be 'very strongly’ distressed by financial worries. Entering this stressor in Block 2 resulted in a substantially decrease of OR for single mothers’ heavy smoking pattern from 2.56 to 2.01, representing a change of 35.3% explained by this stressor. All other stressors, namely ’career situation/unemployment’, ’conflicts with the partner or ex-partner’, ’sole responsibility for the child/ren’ and 'child-rearing difficulties’ failed to reach statistical significance in the multivariate model and thus made no contribution in explaining single mothers’ odds for heavy smoking.

## Discussion

In line with the current state of research [2-6] our findings confirmed higher smoking rates among single as compared to partnered mothers, holding in particular for heavy smoking pattern. Against this background, this study aimed to analyze whether perceived psychosocial stress acts as a mediator and accounts for the relationship between single motherhood and smoking. According to Baron and Kenny [25] psychosocial stressors function as a mediator when they are systematically varied in dependency of the status 'single’ or 'partnered’ mothers (condition 1) and when they in turn significantly effect smoking pattern (condition 2).

### Perceived psychosocial stress in single and partnered mothers

With respect to the first condition, our analysis has shown to be in accordance with many other studies [7-10] indicating that single mothers are at higher risk of poverty and reported considerably higher levels of financial and occupational stress as compared to partnered mothers. We also found single mothers facing higher levels of family-, marital- as well as parenting-related stress and also higher levels of stress related to balancing family and job demands. In this context, Avison et al. [27] pointed out that single mothers are more likely to report that family demands interfere with paid work and vice versa. They see this influence as an example of the ways in which the structure of families and the broader social structure of society interact to create stress among single mothers. Our study confirms these considerations, indicating that single mothers encounter significantly higher stress levels in both social roles: caregiver as well as breadwinner.

### Effects of psychosocial stress on moderate and heavy smoking pattern

We also found that smoking rates increased with single mothers’ levels of perceived psychosocial stress, confirming the second condition of a mediator-effect. In line with previous studies [4,15-17] we found a strong effect of financial worries and job-related stress in particular on the risk of heavy smoking. While the effect of socioeconomic hardship on smoking has been well researched by now, little is known about the role of family- and parenting-related stress on single mothers’ smoking patterns. The few available studies suggest that child care responsibilities and marital stress imply an independent risk of smoking among women [3,14,28,29]. Based on this evidence, we hypothesized that single mothers facing high levels of marital-, parenting- and family-related stress are more likely to smoke than single mothers with lower stress levels. In line with this assumption we found a strong effect of marital conflicts ('conflicts with the partner or ex-partner) on moderate as well as heavy smoking pattern. As expected, also ’sole responsibility for the child/ren’ was associated with moderate as well as heavy smoking pattern. However, scoring high on some other family-related stressors (e.g. household requirements and family demands) showed no significant association with smoking, neither with moderate nor with heavy smoking pattern. Searching for explanations we noticed that stress levels of these stressors increased while smoking rates decreased with higher levels of school education. Hence, there is some evidence that educational attainment moderate the effect of stress on smoking. However, subsequent research is needed to follow this track and empirically substantiate this assumption.

In contrast to a number of studies suggesting a linear relationship between stress level and smoking [13] we found some stressors being associated only with heavy, but not with moderate smoking. This was the case for stress due to 'child-rearing difficulties’, 'little recognition of family work’, 'family member in need of care’, 'conflicts with other family members’ and 'unwanted living alone/isolation’. This finding supports a differentiation between moderate and heavy smoking suggesting that heavy smoking women face not only higher levels of stress, but also different kinds of stress.

Surprisingly, we found significant interaction effects between single motherhood and stress on smoking in a way that among single mothers some family-related stressors were not only irrelevant but rather associated with significantly reduced odds of foremost moderate smoking habits. This may suggest that some family-related stressors such as family demands and household requirements keep single mothers away from smoking. Similarly, we found significant interaction effects with respect to the stressors 'unwanted living alone/loneliness’ and 'little recognition of family work’. These findings demonstrate that the effect of these stressors on heavy smoking only holds in partnered mothers and revealed to be largely irrelevant in explaining single mothers’ heavy smoking pattern. In principle, this finding indicates that the association between stress and smoking differs between single and partnered mothers, pointing to a moderator-effect of single motherhood on the relationship between stress and smoking. This result may provide a starting point for further analysis with the aim of combining mediation and moderation analysis in explaining single mothers’ higher smoking rates.

### Contribution of psychosocial stress in explaining single mothers’ smoking patterns

Our findings revealed that out of the original 13 stressors only two remained relevant in explaining single mothers’ higher odds of smoking. First, this was stress due to 'conflicts with the partner or ex-partner’ which could explain 12% of single mothers’ higher odds for moderate smoking. Financial worries, secondly, turned out to be the most important predictor in explaining single mothers’ heavy smoking pattern. This stressor alone explained 35.3% of their higher odds for heavy smoking. All other stressors failed to reach the conditions for being a mediator or showed no significant effect on smoking in the final model. The association between single motherhood and moderate as well as heavy smoking remained significant after controlling for psychosocial stress. Accordingly, the 'single mother-effect’ on smoking could in part but not completely be attributed to their higher stress exposure.

The finding that the stressors ’career situation/unemployment’, ’conflicts with the partner or ex-partner’, ’sole responsibility for the child/ren’ and 'child-rearing difficulties’ were significantly associated with heavy smoking in the univariate analysis but failed to reach statistical significance in the multivariate model might be attributed to the fact that these stressors are correlated. Indeed, as deeper investigations revealed, these stressors showed significant correlations, reaching Spearman-Rho coefficients ranging from 0.28 to 0.39. We also found significant correlations between these stressors and financial worries (Spearman-Rho coefficients ranging from 0.31 to 0.44), indicating that financial worries also cause increased stress levels, related for example to 'child-rearing difficulties’ and 'conflicts with the partner or ex-partner’. Our results underline the relevance of tackling social disadvantages among single mothers as these efforts not only enhance their material living conditions but also have positive effects on their psychosocial stress level in general. All in all our findings emphasize the need for smoking-cessation programs which not only offer advice on how to quit smoking but also address social and financial stressors that place single mothers at high risk of smoking.

### Limitations

It has to be noted that firm conclusions cannot be drawn on the causal relationship between smoking and stress as the data used were cross-sectional. According to Siahpush et al. [17] it can be assumed that smoking may also create financial stress, particularly among socially disadvantaged single mothers. In addition, we could not adjust for further possible confounders such as urban versus rural residence or physical activity which may contribute to single mothers’ higher smoking rates. It has also to be mentioned that the questionnaire used in this study did not provide information about the smoking history of respondents and their past attempts to quit smoking. As we only asked for current daily stressors we also did not know whether the stressful life situation was acute or chronic. Umberson et al. [30] emphasized the importance of merging stress and life course perspectives to elaborate on the impact of stress on health-related behaviour. This approach is promising for providing deeper insights on trajectories of single mothers’ stress unfolding over the life course and on the question whether their higher stress experience is transitory or stable over time.

## Conclusions

Single mothers’ daily stress experiences are not limited to socioeconomic disadvantages but also include parenting- and family-related strains. The impact of stress on single mothers’ smoking differs between moderate and heavy smoking pattern: while the stressor 'conflicts with the partner or ex-partner’ turned out to be most important in predicting single mothers’ moderate smoking, 'financial worries’ accounted most in explaining their heavy smoking pattern. Our results support a differentiation between moderate and heavy smoking suggesting that heavy more than moderate smoking is mediated by psychosocial stress. Overall, a higher stress exposure explains partly but not completely single mothers’ increased smoking rates.

## Competing interests

We declare that we have no conflicts of interest in the authorship or publication of this contribution. We can exclude any financial and non-financial competing interests.

## Authors’ contribution

SS has made substantial contributions to the concept and design and performed the statistical analysis and wrote the manuscript. MNM participated in the design of the study and helped to draft the manuscript. DN has been involved in revising the manuscript critically for important intellectual content. All authors read and approved the final manuscript.

## Pre-publication history

The pre-publication history for this paper can be accessed here:

http://www.biomedcentral.com/1471-2458/13/1125/prepub

## Supplementary Material

Additional file 1 Questionnaire Parental Stress. Click here for file
