# Supplementary material for: The effect of psychosocial stress on single mothers’ smoking
Source: BMC Public Health. 2013 Dec 5;13:1125. doi: 10.1186/1471-2458-13-1125 (PMC4235024; doi:10.1186/1471-2458-13-1125)
Supplement: Additional file 1 — Questionnaire Parental Stress. [file 1471-2458-13-1125-S1.pdf]

**Questionnaire: Parental Stress**

The list below shows possible burdens of everyday family life. Please state to what extent do you currently feel distressed with respect to the following issues.

| | not at all / not applicable | slightly | moderately | strongly | very strongly |
|---|---|---|---|---|---|
| **Psychosocial stress due to….** | | | | | |
| financial worries | ❑ | ❑ | ❑ | ❑ | ❑ |
| career situation (includes also unemployment) | ❑ | ❑ | ❑ | ❑ | ❑ |
| conflicts with current or former spouse / partner | ❑ | ❑ | ❑ | ❑ | ❑ |
| household requirements | ❑ | ❑ | ❑ | ❑ | ❑ |
| permanent availability and commitment to the family | ❑ | ❑ | ❑ | ❑ | ❑ |
| sole responsibility for the children | ❑ | ❑ | ❑ | ❑ | ❑ |
| a disabled or chronically ill child | ❑ | ❑ | ❑ | ❑ | ❑ |
| child-rearing difficulties / conflicts with the child | ❑ | ❑ | ❑ | ❑ | ❑ |
| a family member in need of care (e.g. spouse, parents) | ❑ | ❑ | ❑ | ❑ | ❑ |
| balancing family and job demands (includes also training / studies) | ❑ | ❑ | ❑ | ❑ | ❑ |
| conflicts with other family members (e.g. parents, parents-in-law) | ❑ | ❑ | ❑ | ❑ | ❑ |
| unwanted living alone / loneliness | ❑ | ❑ | ❑ | ❑ | ❑ |
| little recognition and appreciation of my family work | ❑ | ❑ | ❑ | ❑ | ❑ |
